# Biochemical profiling, prediction of total lipid content and fatty acid profile in oleaginous yeasts by FTIR spectroscopy

**DOI:** 10.1186/s13068-019-1481-0

**Published:** 2019-06-06

**Authors:** Volha Shapaval, Jule Brandenburg, Johanna Blomqvist, Valeria Tafintseva, Volkmar Passoth, Mats Sandgren, Achim Kohler

**Affiliations:** 1 Faculty of Science and Technology, Norwegian University of Life Science, P.O. Box 5003, 1432 Ås, Norway; 2 Department of Molecular Sciences, Swedish University of Agricultural Sciences, BioCenter, Box 7015, 75007 Uppsala, Sweden

**Keywords:** FTIR spectroscopy, Lipids, Oleaginous yeasts, Total lipid content, Lipid profile, Biodiesel

## Abstract

**Background:**

Oleaginous yeasts are considered as a potential lipid source for food, feed and biofuel production. In order to make the yeast-based lipid production environmentally and economically sustainable, there is a need for screening studies in order to find the best yeast lipid producers on different substrates, and to optimize cultivation conditions. Since the target parameter of such screening studies are lipid amounts and profiles, an analytical technique that is able to perform lipid analyses rapidly, reproducible and with high precision is highly desirable. The main objective of this study was to establish the non-invasive high-throughput Fourier transform infrared (FTIR) spectroscopy analysis for the prediction of lipid content and profile in oleaginous yeasts.

**Results:**

High-throughput FTIR spectroscopy allowed characterizing the total biochemical profile of oleaginous yeasts and enabled us to identify strains and substrate(s) providing the highest total lipid content. Some of the yeast strains grown under nitrogen-limiting conditions with glucose/xylose/mixture of glucose and xylose as carbon sources were accumulating lipids with a high proportion of free fatty acids. FTIR spectra were used to predict gravimetric and gas chromatography data by establishing multivariate calibration models. Coefficients of determination (*R*^2^) for calibration models were obtained in a range between 0.62 and 0.92 for predicting lipid content. When using an independent test set, *R*^2^ values between 0.53 and 0.79 were achieved for predicting fatty acid profile. The best spectral region(s) for the prediction of total lipid content was 3100–2800 cm^−1^ combined with 1800–700 cm^−1^, and for prediction of summed saturated (SAT), monounsaturated (MUFA) and polyunsaturated (PUFA) fatty acids: 3100–2800 cm^−1^, 3100–2800 cm^−1^ combined with 1700–1715 cm^−1^ and 3100–2800 cm^−1^ combined with 1800–1715 cm^−1^, respectively. The highest lipid accumulation was observed for strains *Rhodotorula babjevae* DBVPG 8058 on glucose and mixture of glucose and xylose and *Lipomyces starkeyi* CBS 2512 on xylose.

**Conclusions:**

Applying FTIR spectroscopy combined with multivariate data analysis allows performing rapid, non-invasive, reproducible and precise quantitative predictions of total lipid content and lipid profile. It allows also detecting different lipid fractions as triacylglycerols (TAGs) and free fatty acids and evaluating the total biochemical profile of cells. Several yeast strains with high lipid accumulation were identified.

**Electronic supplementary material:**

The online version of this article (10.1186/s13068-019-1481-0) contains supplementary material, which is available to authorized users.

## Background

Oleaginous yeasts are one of the potential alternative sources of lipids for the production of second-generation biodiesel or as ingredients in food and feed [1, 2]. Oleaginous yeasts are able to accumulate lipids in the form of triacylglycerides (TAGs) and store them intracellularly in dynamic organelles—lipid bodies. The lipid accumulation capacity of oleaginous yeasts varies depending on species, strain, substrate and culture conditions, and the total lipid content can account for 20% to 76% of the yeasts’ total biomass [3, 4].

In the last decade, substantial efforts have been taken to develop the “Yeast Lipids-to-Food, Feed and Biodiesel” concept [5–7]. Unfortunately, despite all advantages related to the use of oleaginous yeasts as a source of lipids for biodiesel production, the exploitation of this concept is still at an early developmental stage, and the final product is too expensive to compete with diesel produced from fossil resources or first generation feedstock [8]. The identification and development of oleaginous yeast strains that rapidly accumulate lipids [9, 10], selection of suitable cheap substrates, as well as optimization of the cultivation parameters represent some of the main challenges in developing “Yeast Lipids-to-Food, Feed and Biodiesel” processes. Performing extensive high-throughput screening procedures involving high numbers of yeast lipid producers grown on many different cheap substrates under different cultivation conditions as well as following the kinetics of lipid accumulation requires a method of lipid determination that is rapid, reproducible, and has a high precision for several hundreds of samples in a short period of time. Determination of the lipid profile is particularly important, since yeast lipid producers should be selected for a certain application. For example, it is well known that for the biodiesel production, lipids rich in monounsaturated fatty acids (MUFAs) are preferred, while in cases of animal feed and human food use, lipids rich in polyunsaturated fatty acids (PUFAs) are more valuable [2, 5].

Traditionally, the determination of total lipid content and lipid profile is done by the methods based on lipid extraction using organic solvents and consequent determination of the fatty acid profiles by gas chromatography (GC). Such an approach, due to its time consuming set-up, is not feasible for high-throughput screenings. In addition, lipid extraction procedures involved in the traditional lipid analysis methods require a quite large amount of biomass that makes it difficult to use them for screenings where cultivation of microorganisms is performed in small volumes such as microtiter plate systems.

Currently, there are two main groups of analytical methods that are getting extensively utilized for rapid analysis of microbial lipids: (a) methods based on using lipophilic fluorescent dyes (Sudan black, Nile red, and BODIPY) [11–16] and, (b) methods based on flow cytometry [17]. Both methods are rapid, non-invasive and do not require tedious and time-consuming extraction of lipids, and to some extent have a possibility for high-throughput analysis. However, these methods can only be used for total lipid quantification and are not applicable for the analysis of lipid profiles such as, for example, relative amount of summed saturated (SAT), monounsaturated (MUFA) and polyunsaturated (PUFA) fatty acids. In addition, when it comes to the total lipid content, only a method based on using Nile Red dye has been calibrated and validated against traditional gas chromatography (GC) method [11]. In spite of relatively good validation results for Nile red staining presented in the literature [8], our experience is that Nile red staining is not very accurate for lipid quantification, has low reproducibility and extensive calibration is required for each strain and cultivation conditions (unpublished results).

Recently, vibrational spectroscopy techniques requiring small sample volumes and allowing rapid, non-invasive biochemical fingerprinting of microbial cells have emerged for the analysis and monitoring of lipid accumulation in oleaginous microorganisms [18–24]. Among them, Fourier transform infrared (FTIR) is the spectroscopic technique, which has been actively introduced for high-throughput analysis of microbial lipids [18–22, 25]. FTIR spectroscopy combined with microtiter plate cultivation system was successfully used for high-throughput prediction of total lipid content and lipid profile (SAT, MUFA and PUFA) of accumulated lipids in oleaginous filamentous fungi [18, 20]. FTIR spectroscopy has been successfully calibrated and validated against the traditional GC method [18, 20, 22]. In addition. FTIR spectroscopy was introduced as a rapid method for validation of extraction efficiency of lipid extraction procedures in traditional GC-based methods of lipid analysis [19].

The application of FTIR spectroscopy for the determination of lipid content and profile in oleaginous yeast has been evaluated only in a few research cases: Ami et al. [26] used FTIR for the comparison of lipid accumulation of two oleaginous yeasts and the non-oleaginous species *Saccharomyces cerevisiae.* Deeba et al. [27] and Patel et al. [6] monitored the lipid profile of oleaginous yeasts by FTIR but for extracted lipid samples. To our knowledge, this is the first-ever study reporting the evaluation of FTIR spectroscopy for analysing the total biochemical profile and prediction of total lipid content and lipid profile for a relatively large set of 13 oleaginous yeast strains grown on three different substrates (glucose, xylose and a mixture of glucose and xylose) sampled at different time points of cultivation.

## Methods

### Yeast strains

A set of 13 oleaginous yeast strains from the genera, *Solicoccozyma*, *Lipomyces* and *Rhodotorula*, were used in the study (Table 1). Yeasts were obtained from the CBS-KNAW collection (Utrecht, Netherlands) and the Culture collection at Department of Molecular Sciences, Swedish University of Agricultural Sciences—SLU (Uppsala, Sweden).

**Table 1:** List of oleaginous yeast strains used in the study

Name	Strain number	
*Solicoccozyma terricola*	CBS	4517^a^
*Lipomyces lipofer*	CBS	944^a^
*Lipomyces lipofer*	CBS	5842^a^
*Lipomyces starkeyi*	CBS	1807^a^
*Lipomyces starkeyi*	CBS	2512^a^
*Lipomyces starkeyi*	CBS	7544^a^
*Rhodotorula babjevae*	CBS	7808^a^
*Rhodotorula babjevae*	CBS	7809^a^
*Rhodotorula babjevae*	DVBPG	8058^b^
*Rhodotorula glutinis*	CBS	20^a^
*Rhodotorula glutinis*	CBS	5805^a^
*Rhodotorula graminis*	CBS	3043^a^
*Rhodotorula toruloides*	CBS	14^a^

^a^CBS, Utrecht, The Netherlands

^b^Industrial Yeast Collection, Perugia, Italy

### Media and growth conditions

All yeast strains were stored at − 80 °C in 50% v/v glycerol and pre-grown on YPD agar plates (20 g/L glucose, 20 g/L peptone, 10 g/L yeast extract and 16 g/L agar) for 3 to 4 days at 25 °C. Thereafter, the plates were kept in a refrigerator and re-streaked monthly. The pre-cultures (P) were prepared in 50 mL of YPD media (containing 20 g/L glucose, 20 g/L peptone and 10 g/L yeast extract) in 250-mL baffled shake flasks by cultivation for 2 to 3 days at 25 °C on an orbital shaker at 125 rpm. The pre-cultures (P) were used as inoculum for cultivation on nitrogen-limited YNB medium with different carbon sources.

For FTIR spectroscopy, lipid extraction and gas chromatography (GC) analysis, yeast strains were cultivated in a nitrogen-limited YNB-based medium containing 1.7 g/L Yeast Nitrogen Base (YNB) without ammonium sulphate and amino acids (DifcoTM, Becton–Dickinson and Company, USA), 0.75 g/L yeast extract, 2 g/L ammonium sulphate and a 0.2 M phosphate buffer pH 6. As a carbon source, three different sugar solutions, glucose (G) or xylose (X) or a mixture (M) of both (1:1) in a final total sugar concentration of 70 g/L, were used. The cultivation in YNB was done at 25 °C for 120 h on an orbital shaker at 125 rpm. Samples from YNB cultivations taken after 120 h (G/X/M) and samples from pre-cultures (P) were analysed by both FTIR spectroscopy, lipid extraction and gas chromatography.

### Preparation of yeast biomass for FTIR spectroscopy

A small portion of the cell suspensions was transferred from the shake flasks into Eppendorf tubes, and yeast biomass was washed three times with NaCl solution (1 g/L) to remove the remaining growth medium. After the last washing step, approximately 50 μL of cell suspension remained in the Eppendorf tubes and was further used for FTIR analysis.

### High-throughput FTIR spectroscopy

FTIR spectroscopy analysis of washed yeast biomass was performed using the High Throughput Screening eXTension (HTS-XT) unit coupled to the Vertex 70 FTIR spectrometer (both from Bruker Optik, Germany) in transmission mode. Of each cell suspension, 8 µL was transferred to an IR-light-transparent silicon 384-well microplate (Bruker Optik, Germany). Samples were dried at room temperature for 45 min before FTIR analysis. The spectra were recorded in the region between 4000 and 500 cm^−1^ with a spectral resolution of 6 cm^−1^ and an aperture of 5.0 mm. For each spectrum, 64 scans were averaged.

### Lipid extraction and GC analysis

Lipids were quantified gravimetrically after extraction by a modified Folch-method. Fatty acid profiles were determined by GC analysis, after conversion of the lipids to fatty acid methyl esters [28, 29].

### Thin layer chromatography (TLC) for analysis of lipid classes

The determination of lipid classes was done according to Olsen and Henderson [30] with slight modification. Extracted lipid samples were diluted to a concentration of 1 g/L in hexane, and 5 μL of each solution (5 µg of lipids) was applied with a CAMAG TLC Sampler ATS4 (Camag Switzerland) 2 cm from the base edge of the TLC plate (pre-coated with silica gel TLC plates. 20 × 10 cm; Silicagel 60; 0.20 mm layer, Merck, Darmstadt. Germany) in 2 mm bands with an application speed of 250 nL/s. Nitrogen was used as a spray gas. All samples were applied in duplicate, and the distance between tracks was 9.8 mm. Separation of the lipid classes was executed with a CAMAG Automatic Developing Chamber 2 (ADC 2) (Camag Switzerland). For the separation of the lipid classes. hexane:diethyl ether:acetic acid (85:15:2; v/v/v) was used as a mobile phase. After the separation procedure, plates were dipped in a solution of 3% cupric acetate in 8% phosphoric acid and then charred for 20 min at 140 °C. Quantitative analysis of the separated lipid classes was done by scanning the plates using a CAMAG TLC Scanner 3 (Camag, Switzerland). The scanning was performed at a speed of 20 mm/s and a data resolution of 100 μm/step, with a slit dimension of 6.00 × 0.45 mm at a wavelength of 350 nm. Identification of the lipid classes was performed by comparison with external standards (TLC 18-4A, Nu-Chek Prep, Elysian, USA; Ergosterol, PHR1512, Sigma-Aldrich, Sweden). For the data processing, Savitsky-Golay with seven smoothing points was used, and baseline and peak corrections were done manually where it was necessary.

### Experimental design

FTIR analysis of yeast strains cultivated for 120 h on glucose (G), xylose (X) and mixture of both (1:1) (M), and 3 days’ cultivated pre-cultures (P) were done in two biological replicates performed as independent experiments on separate days. Three FTIR technical replicates for each biological replicate were prepared.

### Multivariate data analysis

FTIR spectra (4000–500 cm^−1^) were preprocessed by two preprocessing strategies depending on the calibration model to be developed. The pre-processing approach is in line with the pre-processing strategies developed for lipids in the Ref. [31]: (a) for total lipid prediction: FTIR spectra were corrected by Extended Multiplicative Scatter Correction (EMSC) with linear and quadratic components followed by averaging of biological and technical replicates. In order to optimize the baseline estimations in the EMSC, spectral inactive regions were up-weighted in the EMSC modelling; (b) for SAT, MUFA and PUFA predictions: FTIR spectra were preprocessed by transforming to second derivative form with the Savitzky-Golay (S-G) method (second degree polynomial, 9 or 15 windows size), followed by EMSC with linear and quadratic components [31]. Total biochemical profile was evaluated by (a) visual inspection of line plots of spectra preprocessed and by (b) Principal Component Analysis (PCA) of spectra preprocessed. Partial Least Square Regression (PLSR) was used to develop fatty acid profile prediction models by calibrating FTIR spectra preprocessed by (a) and (b) against gravimetric and GC reference data. FTIR spectra were used as X variables (or predictors), and gravimetric and GC fatty acid data (Additional file 1: Table S1) as Y variables (or responses). For PLSR modelling, FTIR data were divided into calibration (52 samples) and test set (18 samples). A leave-one-biological replicate-out cross-validation (CV) scheme was used to optimize the calibration models, and the optimal number of PLS factors was estimated by visually inspecting the Root-Mean-Square-Error of CV (RMSECV) [23–25].

The following spectral regions were examined for lipid profile predictions: (a) 3100–2800 cm^−1^, (b) 3100–2800 cm^−1^ combined with 1800–700 cm^−1^, (c) 3100–2800 cm^−1^ combined with 1800–1700 cm^−1^, (d) 3100–2800 cm^−1^ combined with 1700–1715 cm^−1^, (e) 3100–2800 cm^−1^ combined with 1800–1715 cm^−1^, (f) 1800–1700 cm^−1^, (g) 1800–700 cm^−1^. The regions 3100–2800 cm^−1^ and 3100–2800 cm^−1^ combined with 1800–700 cm^−1^ were examined for the prediction of the total lipid content.

## Results

### Total biochemical profile of oleaginous yeasts by FTIR spectroscopy

**Fig. 1:**
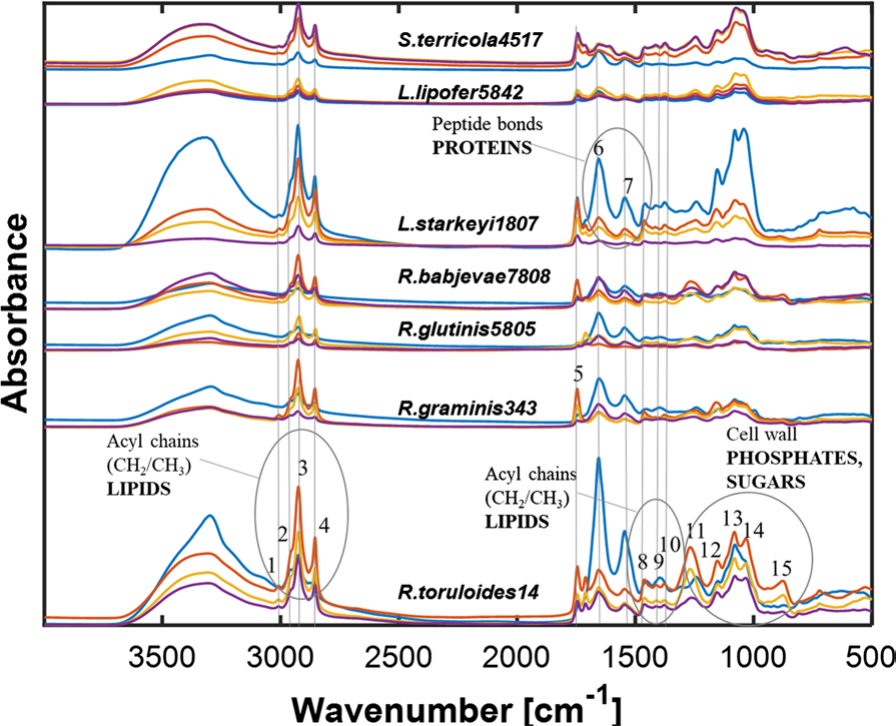
EMSC corrected, according to the preprocessing strategy (a), and consequently averaged FTIR spectra of seven biodiesel relevant oleaginous yeasts cultivated in pre-culture medium—P (blue), YNB medium containing glucose—G (red), xylose—X (orange) and mixture of glucose and xylose (1:1)—M (purple). Peaks assignments are presented in Table 2

**Fig. 2:**
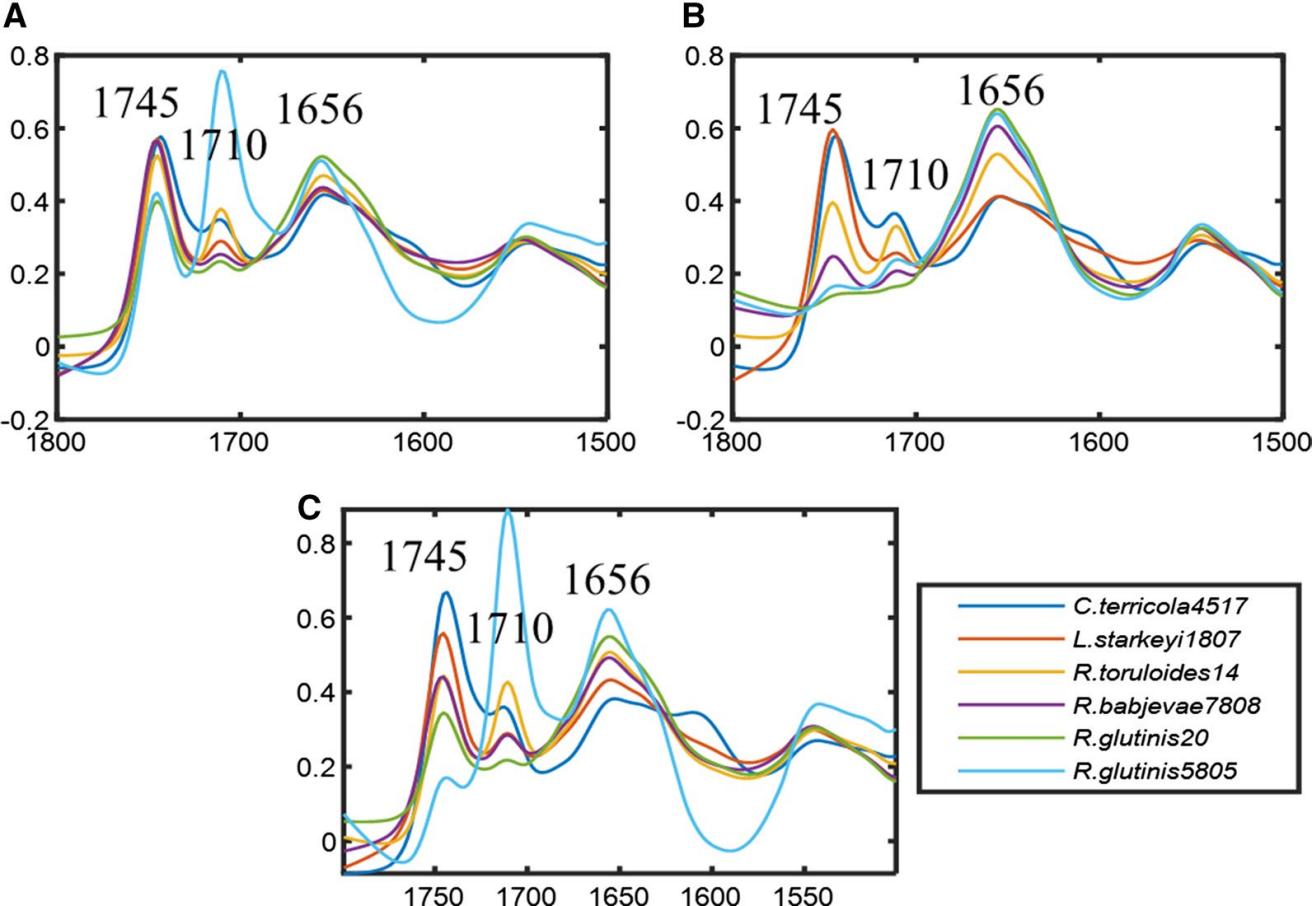
EMSC corrected, according to the preprocessing strategy (a), and consequently averaged FTIR spectra of six biodiesel relevant oleaginous yeasts: *Solicoccozyma terricola* CBS 4517 (blue), *Lipomyces starkeyi* CBS 1807 (red), *Rhodotorula toruloides* CBS 14 (orange), *Rhodotorula babjevae* CBS 7808 (purple), *Rhodotorula glutinis* CBS 20 (green) and *Rhodotorula glutinis* CBS 5805 (light blue) cultivated in YNB medium containing glucose—G (**A**), xylose—X (**B**) and mixture of glucose and xylose (1:1)—M (**C**)

**Fig. 3:**
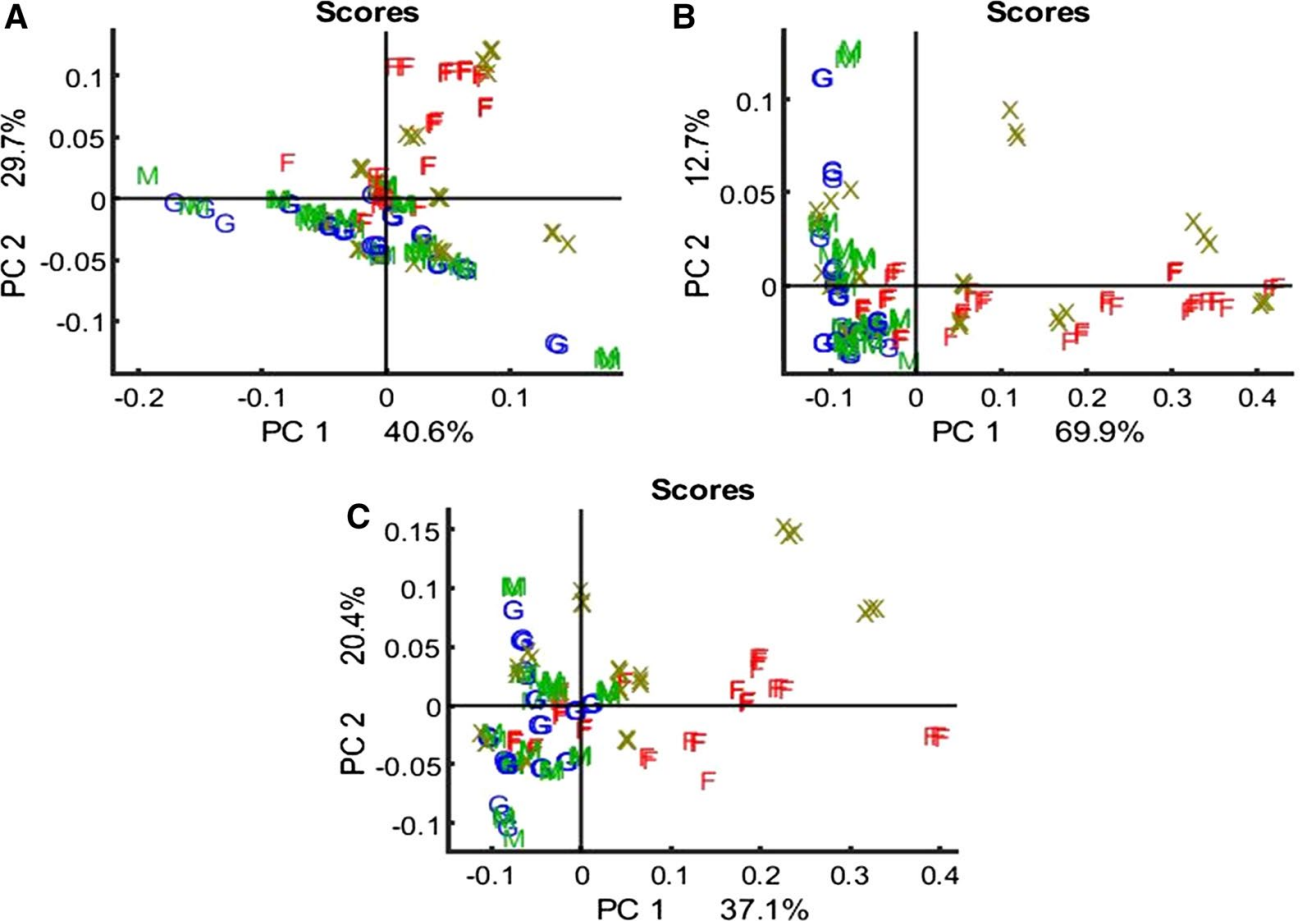
PCA score plots of EMSC corrected, according to the preprocessing strategy (b). FTIR spectra for lipid region 3100–2800 cm^−1^ combined with 1800–1700 cm^−1^ (**A**), protein region 1700–1500 cm^−1^ (**B**), and carbohydrate region 1200–700 cm^−1^ (**C**)

Thirteen biodiesel relevant oleaginous yeast strains were cultivated under different conditions, i.e. 3 days of cultivation on lipid-rich pre-culture medium (P) and 120 h of cultivation on nitrogen-limited media with three different C-sources (G, X, and M). After growth, Fourier transform infrared spectroscopy (FTIR) analysis was performed on all samples resulting in a set of 504 FTIR spectra. The FTIR spectra showed different total biochemical profiles, depending on the C-source in the growth medium and time of the cultivation. The evaluation of the total biochemical FTIR profile was performed by visual inspection of EMSC-corrected and subsequently averaged FTIR spectra (see preprocessing (a) shown in Figs. 1 and 2). Clustering of samples was studied by inspecting score plots from the PCA analysis of derivative forms of FTIR spectra and EMSC-corrected spectra (see preprocessing (b) shown in Fig. 3).

The total biochemical FTIR profiles of yeasts grown in pre-culture medium (P), glucose (G), xylose (X) and mixture of glucose and xylose (1:1) (M) are represented by the sets of characteristic peaks for lipids in the spectral regions 3020–2800 cm^−1^, 1800–1700 cm^−1^, 1500–1300 cm^−1^, 1100–1200 cm^−1^ and 800–700 cm^−1^, for proteins in the spectral region 1700–1500 cm^−1^, carbohydrates in the spectral region 1200–800 cm^−1^ and polyphosphates, phospholipids and nucleid acids in the spectral region 1300–1200 cm^−1^ (Fig. 1, Table 2) [20]. The biochemical lipid FTIR profiles (Fig. 1, Table 2) of the studied yeasts are represented by the following main characteristic peaks: 3010 cm^−1^ representing =C–H stretch in fatty acids of TAGs; 2955 cm^−1^ and 1380 cm^−1^ representing stretching CH_3_ of acyl chains in fatty acids of TAGs; 2925 cm^−1^, 2850 cm^−1^ and 725 cm^−1^ representing stretchings CH_2_ of acyl chains in fatty acids of TAGs; and 1745 cm^−1^ representing C=O stretching in ethyl esters and indicating the total lipid content in the cell.

**Table 2:** Tentative peak assignment of spectral bands in FTIR spectra of fungi [20]

Peak nr.	Frequency (cm^−1^)	Peak assignment	Main biomolecules
1	3010	=C–H stretching	Lipid
2	2955	C–H assymetric stretching of –CH_3_	Lipid
3	2925	Stretching of > CH_2_ of acyl chains (assymetric)	Lipid
4	2850	Stretching of CH_2_ of acyl chains (symetric)	Lipid
5	1745	C=O stretching	Lipid
6	1680–1640	Amide I band (C=O stretching)	Protein
7	1580–1520	Amide II (CONH bending)	Protein
8	1465	CH_2_ deformtion	Lipid
9	1410	Amide III band (C–N stretching)	Protein
10	1380	CH_3_ bending	Lipid
11	1240–1265	P=O stretching (assymetric) of > PO_2_ phosphodiesters	Polyphosphate, phospholipid
12	1155	C–O–C stretching	Lipid
13	1080	P O stretching (symetric) of > PO_2_	Polyphosphate, phospholipid
14	900–1200	C–O and C–C stretching, C–O–H and C–O–C deformation	Carbohydrate
15	875	P–O–P stretching	Polyphosphate, phospholipid
16	725	CH_2_ deformation	Lipid

The FTIR spectra of yeasts grown on glucose (G) and on a mixture of glucose and xylose (M) show that, for most of the yeast strains, the absorbance values at the lipid peak at 1745 cm^−1^, indicating the total lipid content in the yeast biomass, exceed the absorbance values of protein peaks (1700–1500 cm^−1^) and polysaccharide peaks (1200–900 cm^−1^) (Fig. 1). For many yeast strains cultivated on xylose (X), the absorbance values at the lipid peak at 1745 cm^−1^ were below the absorbance values of protein (1700–1500 cm^−1^) and polysaccharide peaks (1200–900 cm^−1^) (Fig. 1). The total biochemical FTIR profiles of yeasts cultivated in the pre-culture medium show that several yeast strains have strong absorbance values at 1745 cm^−1^ peak, but for all yeasts, the lipid peak was lower than protein peaks (1700–1500 cm^−1^) and polysaccharide peaks (1200–900 cm^−1^) (Fig. 1). The absorbance at 3010 cm^−1^, indicating the level of TAGs unsaturation, was observed to appear with strongly varying absorbance values for all studied yeasts cultivated on all growth media for different time points (Fig. 1). In addition, for some yeasts, for example, *S. terricola* CBS 4517, *L. starkeyi* CBS 1807, *R. toruloides* CBS 14, *R. babjevae* CBS 7808, *R. glutinis* CBS 20, *R. glutinis* CBS 5805, grown on glucose (G), xylose (X) and a mixture of glucose and xylose (M), absorbance values at 1710 cm^−1^ were observed, indicating the presence of significant amounts of free fatty acids in the accumulated lipids (Fig. 2). Interestingly, for the yeast strain *R. glutinis* CBS 5805, the absorbance at 1710 cm^−1^ was higher than the absorbance at 1745 cm^−1^ for samples grown on glucose (G) and a mixture of glucose and xylose (M) (Fig. 2). This may indicate that share of free fatty acids is high compared to the share of triacylglycerols (TAGs) in the accumulated lipids.

The PCA analysis of derivated and EMSC-corrected FTIR spectra of three spectral regions, lipid (3100–2800 cm^−1^ combined with 1800–1700 cm^−1^), protein (1700–1500 cm^−1^) and carbohydrate (1200–700 cm^−1^), showed that yeast strains cultivated in the pre-culture medium (P) have very different lipid, protein and carbohydrate profiles due to both phylogenetic differences and growth media (Fig. 3). All the studied yeast strains cultivated in glucose (G), a mixture of glucose and xylose (M), and some of the yeast strains cultivated on xylose (X), show very similar protein and carbohydrate profiles (Fig. 3b, c). The lipid profiles of yeasts cultivated on glucose (G) and a mixture of glucose and xylose (M) show a certain similarity (Fig. 3a), while the lipid profiles of yeasts cultivated on xylose look different compared to G and M (Fig. 3a).

### Total lipid content and lipid profile by traditional gas chromatography (GC)

The total lipid content of the oleaginous yeasts grown in pre-culture medium (P) was in a range of 7.6–24.2% of cell dry weight (Table 3). At the end of cultivation in the nitrogen-limited media containing glucose (G), xylose (X) and a mixture of glucose and xylose (M), the lipid contents were in order of 40%, 24%, and 32%, respectively, with strong variations between different strains. The lipid extraction total lipid results are confirmed by the FTIR results, since FTIR prediction models for the total lipid content show high correlation between lipid extraction and FTIR data (Table 4). Generally, not all yeast strains showed good lipid accumulation when grown on xylose-based media (X) (Additional file 1: Table S1), especially the yeast strains *R. glutinis* 5805, *R. glutinis* 20*, R. toruloides* 14 and *R. graminis* 3043. The highest intracellular lipid content was obtained by *L. starkeyi* 2512 reaching 54.22% when grown on glucose (G), 52.19% when grown on xylose (X) and 54.29% when grown on a sugar mixture (M).

**Table 3:** Range, mean (M), standard deviation (STD) of abundant fatty acids, summed of saturated (SAT) monounsaturated (MUFA), polyunsaturated (PUFA) fatty acids and total fat content in oleaginous yeast samples cultivated in pre-culture medium (P) and on nitrogen-limited medium containing glucose (G), xylose (X) or a mixture of glucose and xylose (M)

Lipid profile	% of total amount			
	High nitrogen glucose-based media (P)	Low nitrogen glucose-based media (G)	Low nitrogen xylose-based media (X)	Low nitrogen glucose and xylose-based media (M)
SAT	8.33–39.13	22.88–51.68	15.54–51.22	21.19–50.37
MUFA	46.19–82.13	44.52–71.9	28.4–72.07	44.37–70.3
PUFA	2.04–16.24	2.82–18.58	3.12–56.06	2.64–25.69

The values were determined by using GC data

**Table 4:** Prediction of total lipid content in yeast biomass by FTIR spectroscopy, using partial least square regression (PLSR) analysis

Spectral region (cm^−1^)	${{\text{RMSE}}_{{{\text{CV}}}}} ^{{\text{a}}}$ (*N* = 149)	RMSE_Test_ (*N* = 56)	${R^{2} {_{{{\text{CV}}}}}} ^{{\text{b}}}$ (*N* = 149)	*R* ^2^_Test_ (*N* = 56)	*N*Factors^c^
3100–2800. 1800–700	4.12	19.00	0.92	0.67	10

*N* is the number of samples in the dataset

^a^RMSE—root mean squared error of cross-validation in calibration (RMSE_CV_) and test set (RMSE_Test_)

^a^*R*^2^—coefficient of determination for cross-validation in calibration (*R*^2^_CV_) and test set (*R*^2^_Test_)

^c^*N*Factors—number of factors used in PLSR models

The highest intracellular lipid content obtained by using FTIR prediction was found in *L. starkeyi* 2512 reaching 54.22% when grown on glucose, 52.19% when grown on xylose and 54.29% when grown on a sugar mixture. The second highest intracellular lipid content was obtained by *R. babjevae* DBVPG 8058 with 54.4% on glucose and 51% on the sugar mixture. However, after growth on xylose, the lipid content was only 23.61%. In general, the *Lipomyces* strains and *S. terricola* CBS 4517 were apparently not influenced by the carbon source and reached similar lipid contents independent of the sugar in the medium.

In the fatty acid profiles of all tested oleaginous strains, the dominating fatty acids were C18:1 with proportions 40–80% of total lipid content, and C16:0 with about 10–40%, followed by C 18:2 (1–40%), C18:0 (1–13%) and C16:1 (0–15%). These results are in line with the prediction results for SAT, MUFA and PUFA from the FTIR spectra (Tables 5, 6, 7), showing high correlation between GC and FTIR. Generally, there were high variations of the fatty acid profiles for the different strains. The fatty acid profiles differed only slightly depending on the carbon source present in the media. Under nitrogen-limiting conditions, we observed an increased amount of C16:0 and a decreased amount of C18:1 compared to the pre-culture (P) condition. The amount of PUFAs were higher in the basidiomycetous red yeasts (*R. glutinis. R. toruloides* and *R. graminis*), compared to the ascomycotous yeasts (*Lipomyces starkeyi*. *Lipomyces lipofer*. *Solicoccozyma terricola*) (Additional file 1: Table S1).

**Table 5:** Prediction of summed saturated fatty acids (SAT) content in yeast biomass by FTIR spectroscopy, using partial least square regression (PLSR) analysis

Spectral region (cm^−1^)	${{\text{RMSE}}_{{{\text{CV}}}}} ^{{\text{a}}}$ (*N* = 125)	RMSE_Test_ (*N* = 56)	${R^{2} {_{{{\text{CV}}}}}} ^{{\text{b}}}$ (*N* = 125)	*R* ^2^_Test_ (*N* = 56)	*N*Factors^c^
3100–2800	4.50	6.70	0.78	0.73	11
3100–2800. 1800–700	3.97	12.74	0.83	0.15	14
3100–2800. 1800–1700	5.46	8.06	0.67	0.50	4
3100–2800. 1700–1715	4.64	5.68	0.77	0.70	12
3100–2800. 1800–1715	4.41	5.70	0.79	0.69	17
1800–1700	6.91	13.28	0.51	0.02	5
1800–700	5.51	11.58	0.67	0.23	5

*N* is the number of samples in the dataset

^a^RMSE—root mean squared error of cross-validation in calibration (RMSE_CV_) and test set (RMSE_Test_)

^b^*R*^2^—coefficient of determination for cross-validation in calibration (*R*^2^_CV_) and test set (*R*^2^_Test_)

^c^*N*Factors—number of factors used in PLSR models

**Table 6:** Prediction of summed monounsaturated fatty acids (MUFA) content in yeast biomass by FTIR spectroscopy, using partial least square regression (PLSR) analysis

Spectral region (cm^−1^)	${{\text{RMSE}}_{{{\text{CV}}}}} ^{{\text{a}}}$ (*N* = 125)	RMSE_Test_ (*N* = 56)	${R^{2} {_{{{\text{CV}}}}}} ^{{\text{b}}}$ (*N* = 125)	*R* ^2^_Test_ (*N* = 56)	*N*Factors^c^
3100–2800	4.99	4.34	0.79	0.79	11
3100–2800. 1800–700	8.85	15.42	0.37	0.29	10
3100–2800. 1800–1700	6.54	11.02	0.65	0.37	9
3100–2800. 1700–1715	5.30	4.14	0.76	0.77	12
3100–2800. 1800–1715	7.49	8.18	0.62	0.55	14
1800–1700	9.76	22.31	0.27	0.10	5
1800–700	9.62	15.11	0.27	0.02	5

*N* is the number of samples in the dataset

^a^RMSE—root mean squared error of cross-validation in calibration (RMSE_CV_) and test set (RMSE_Test_)

^b^*R*^2^—coefficient of determination for cross-validation in calibration (*R*^2^_CV_) and test set (*R*^2^_Test_)

^c^*N*Factors—number of factors used in PLSR models

**Table 7:** Prediction of summed polyunsaturated fatty acids (PUFA) content in yeast biomass by FTIR spectroscopy, using partial least square regression (PLSR) analysis

Spectral region (cm^−1^)	${{\text{RMSE}}_{{{\text{CV}}}}} ^{{\text{a}}}$ (*N* = 125)	RMSE_Test_ (*N* = 56)	${R^{2} {_{{{\text{CV}}}}}} ^{{\text{b}}}$ (*N* = 125)	*R* ^2^_Test_ (*N* = 56)	*N*Factors^c^
3100–2800	4.58	2.43	0.73	0.75	7
3100–2800. 1800–700	7.50	7.80	0.29	0.15	4
3100–2800. 1800–1700	4.54	5.89	0.74	0.46	11
3100–2800. 1700–1715	4.72	2.48	0.71	0.70	8
3100–2800. 1800–1715	4.50	4.46	0.74	0.53	11
1800–1700	7.24	13.79	0.34	0.25	6
1800–700	7.35	5.73	0.31	0.20	4

*N* is the number of samples in the dataset

^a^RMSE—root mean squared error of cross-validation in calibration (RMSE_CV_) and test set (RMSE_Test_)

^b^*R*^2^ number of factors used in PLSR models

^c^NFactors—number of factors used in PLSR models

### Lipid classes by thin layer chromatography (TLC)

In order to study further the appearance of free fatty acids in some of the yeast strains as manifested by FTIR spectroscopy, we performed thin layer chromatography (TLC) with lipids of yeast strains showing a free fatty acid peak at 1710 cm^−1^ in FTIR spectra. The TLC analysis revealed phospholipids. Monoacylglycerols, 1,2- and 1,3-diacylglycerols, sterols (ergosterol), free fatty acids and triacylglycerols (TAGs) (Additional file 1: Table S2).

After cultivation in nitrogen-limited media (G/X/M), which led to an increased lipid content, we observed that the TAGs were the dominating lipid class with a proportion of 60% to 75% of all lipids (Additional file 1: Table S2). Interestingly, we found a high amount of free fatty acids in all pre-cultures (P) with a proportion of 25% up to 41% of total lipids (Additional file 1: Table S2). Even after growth under nitrogen limitation, the free fatty acids represented the second largest fraction of lipids with shares between 6.3 and 27.8% (Additional file 1: Table S2).

### Prediction of total lipid content in oleaginous yeast by FTIR spectroscopy

PLSR was used to establish a prediction model for total lipid content as described in “Methods”. By testing different FTIR spectral regions for the prediction of total lipid content, it was observed that only combination of region 3100–2800 cm^−1^ with 1800–700 cm^−1^ provided a good correlation with the lipid extraction gravimetric data. The coefficient of determination for this region for calibration and test set was *R*^2^_CV_ = 0.92 and *R*^2^_Test_ = 0.67, respectively (Table 4).

### Prediction of lipid profile in oleaginous yeasts by FTIR spectroscopy

**Fig. 4:**
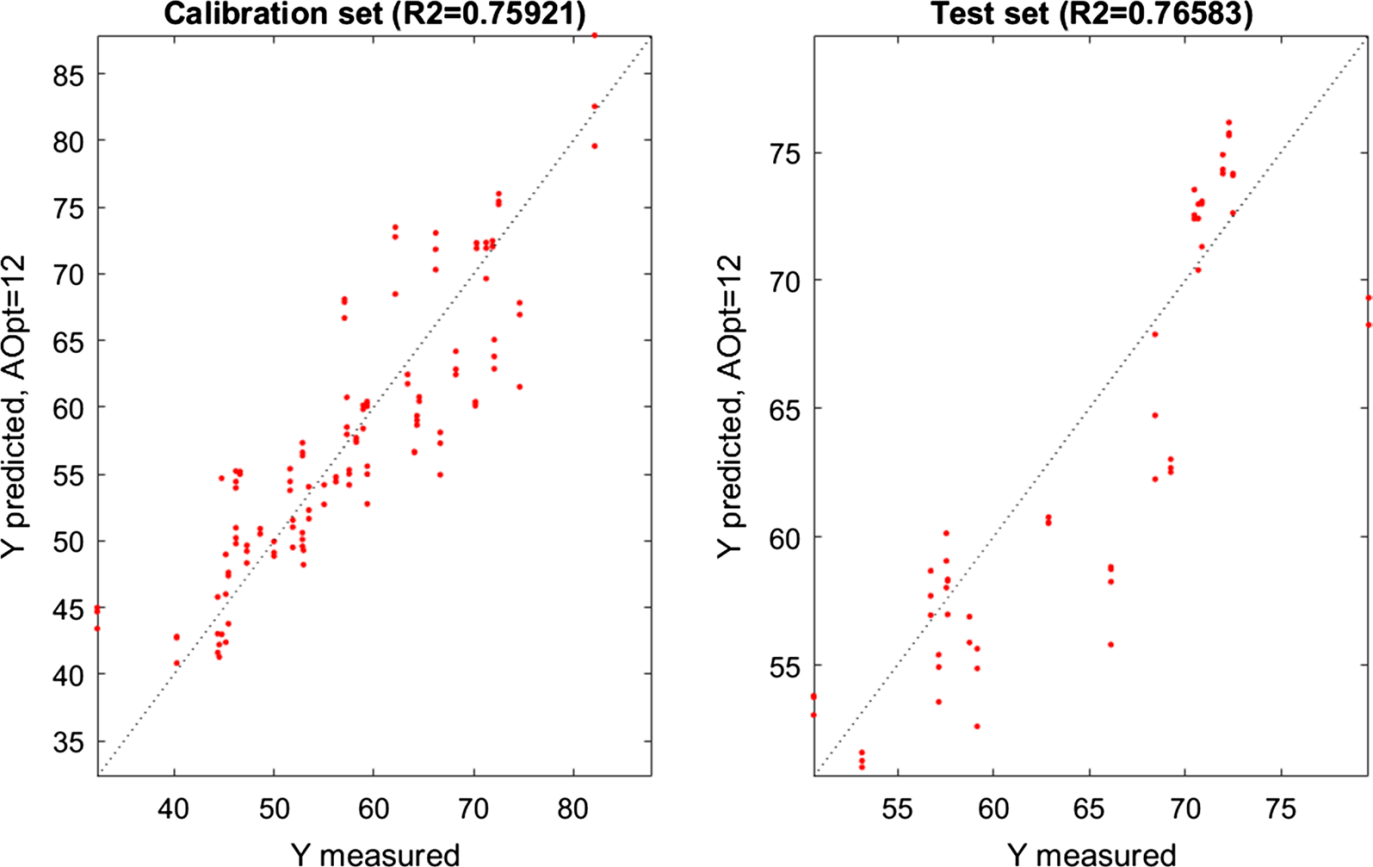
Scatter plots of FTIR and GC data for calibration and test set for prediction of monounsaturated fatty acids (MUFA)

Several FTIR spectral regions were evaluated for the prediction of the lipid profile (SAT, MUFA and PUFA) in oleaginous yeasts by FTIR spectroscopy (Tables 5, 6, 7, Fig. 4). For the prediction of SAT, all evaluated spectral regions showed good correlation between FTIR and GC data in PLSR models of the calibration set, with a coefficient of determination for cross-validation (CV) *R*^2^_CV_ between 0.51 and 0.83 (Table 5). When an independent test set of FTIR data was used for SAT, coefficients of determination *R*^2^_Test_ were obtained in a range of 0.5 to 0.73 (Table 5) for four spectral regions: (a) 3100–2800 cm^−1^, related to CH_2_ and CH_3_ stretching in fatty acids of TAGs; (b) 3100–2800 cm^−1^ combined with 1800–1700 cm^−1^, related to C=O ester and –C=O bond in free fatty acids; (c) 3100–2800 cm^−1^ combined with 1700–1715 cm^−1^, related to C=O ester only; and (d) 3100–2800 cm^−1^ combined with 1800–1715 cm^−1^, related to –C=O bond in free fatty acids only. Calibration results for the prediction of MUFA and PUFA contents achieved coefficients of determination of CV, *R*^2^_CV_ in the range between 0.62 and 0.79 for the spectral regions 3100–2800 cm^−1^, 3100–2800 cm^−1^ combined with 1800–1700 cm^−1^, 3100–2800 cm^−1^ combined with 1700–1715 cm^−1^ and 3100–2800 cm^−1^ combined with 1800–1715 cm^−1^. Almost all of them, except for spectral region 3100–2800 cm^−1^ combined with 1800–1700 cm^−1^, performed well in validation with the test set and *R*^2^_Test_ was obtained in the range 0.53–0.79.

### Detecting cross-correlation between total lipid content and lipid profile

Reference lipid extraction gravimetric data and gas chromatography data were used to investigate the cross-correlation between total lipid content and SAT, MUFA and PUFA (Table 7). Generally, a very low cross-correlation was observed, where total lipid content was not correlated to any of summed fatty acids, while SAT and MUFA showed some correlation (Table 7).

## Discussion

High-throughput Fourier transform Infrared (FTIR) spectroscopy has in the past years been extensively employed for analysis and quantitative prediction of total lipid content and fatty acid profiles in oleaginous filamentous fungi [18, 20, 32]. Here we present the first-ever study of lipid content and lipid profile by FTIR spectroscopy for a variety of oleaginous yeast strains grown on different carbon sources.

FTIR spectroscopy is a highly versatile tool for the analysis of lipids in microbial cells and can be exploited for different purposes, depending on the type of the information and the precision level required: (i) visual inspection of FTIR spectra provides information about the overall biochemical composition of microbial cells; (ii) multivariate analysis of FTIR spectra, as for example PCA, on different spectral regions provides information about variability of samples and main biochemical cell components (lipids, proteins and polysaccharides); and (iii) building prediction models by calibrating FTIR against reference lipid extraction and GC data provides quanititative estimation of total lipid content and fatty acid profile.

Visual inspection of different spectral regions and peaks of FTIR spectra of the oleaginous yeasts from this study grown on different carbon sources showed that most of the yeast strains had a relatively high lipid accumulation already in pre-culture (P) medium. We observed higher absorbance peaks related to lipids than those related to proteins in cases when yeasts were cultivated under nitrogen-limiting conditions, in media containing glucose (G) or a mixture of glucose and xylose (M). The main stimulator of the high lipid accumulation was obviously glucose since FTIR spectra of yeasts grown on the media with only xylose (X) as a carbon source showed higher absorbance values for protein-related peaks than for some of the lipid-related peaks.

In addition, FTIR spectra for some yeast strains grown under nitrogen-limiting conditions showed absorbance values at a peak at 1710 cm^−1^ indicating the presence of free fatty acids in a considerable amount. These results were confirmed by thin layer chromatography (TLC) where relatively high concentrations of free fatty acids were detected. Free fatty acids were the second largest fraction of lipids accumulated in yeast strains grown on different carbon sources under nitrogen-limiting conditions and having a peak at 1710 cm^−1^ in FTIR spectra. While TLC detected the highest proportion of free fatty acids for the same yeast strains grown in pre-culture (P) medium, this observation was not confirmed by FTIR data. The detection of free fatty acids in FTIR spectra of yeasts grown under nitrogen-limiting conditions (G/X/M) and not in pre-culture (P) grown yeasts could be related to the fact that the total lipid content in pre-culture grown yeasts was much lower and therefore the amount of free fatty acids, even though it was present in a high proportion with respect to the total lipid content was beyond the FTIR detection limit.

PCA of FTIR data showed that pre-culture grown yeasts have very different biochemical profiles of lipids, proteins and polysaccharides compared to yeasts grown under nitrogen-limiting conditions with glucose, xylose and a mixture of both. Yeasts grown under nitrogen-limiting conditions with different carbon sources have very similar biochemical profiles of proteins and carbohydrates, while lipid profiles of yeasts grown on xylose show biochemically different FTIR spectra compared to yeasts grown on glucose and mixture of glucose and xylose.

FTIR spectroscopy can also be used as a highly reproducible and precise method for quantitative prediction of total lipids content and lipid profile. By applying PLSR, calibration models between FTIR and GC data were developed, and further validated for the prediction of total lipid content and fatty acid profiles (SAT, MUFA and PUFA). FTIR data showed a good correlation with gravimetric data and GC data for the prediction of total lipid content and lipid profiles revealing coefficients of determination of cross-validation *R*^2^_CV_ ranging from 0.62 to 0.92 for calibration models and *R*^2^_Test_ from 0.53 to 0.79 when independent test sets were applied. As a part of optimization of prediction models for summed fatty acids we evaluated several spectral regions, and it was observed that three spectral regions 3100–2800 cm^−1^, 3100–2800 cm^−1^ combined with 1700–1715 cm^−1^ and 3100–2800 cm^−1^ combined with 1800–1715 cm^−1^ showed the best results. For the prediction of total lipid content, we used only one spectral region 3100–2800 cm^−1^ combined with 1800–700 cm^−1^. This is because the increase in lipid concentration influences the change in other biochemical components of the cell such as proteins and carbohydrates, and thus it is necessary to consider whole biochemical information when predicting total lipid content.

To summarize, the screening of oleaginous biodiesel relevant yeasts with FTIR spectroscopy and gas chromatography showed the highest lipid accumulation for strains *Rhodotorula babjevae* DVPG 8058 on glucose and mixture of glucose and xylose and *Lipomyces starkeyi* 2512 on xylose. Significant amounts of free fatty acids in accumulated lipids were detected for *Rhodotorula glutinis* 5805 grown on all types of tested sugars. Investigating gas chromatography data did not reveal any cross-correlation between total lipid content and fatty acid profile, and low cross-correlation was observed between SAT and MUFA fatty acids.

## Conclusions

The study showed that FTIR spectroscopy data have a high correlation with the reference lipid extraction and gas chromatography data. Therefore, high-throughput FTIR spectroscopy is a potent technique for robust and precise analysis of lipids in oleaginous microorganisms in extensive screening studies. In this study, it has been shown that FTIR spectroscopy data can be used differently —from general inspection and evaluation of total biochemical profile of cells to quantitative prediction of total lipid content and lipid profile. During the study, several oleaginous yeast strains with high lipid accumulation were identified. These strains could be considered as promising lipid producers for further development of a “Yeast Lipids-to-Food, Feed and Biodiesel” concept (Table 8).

**Table 8:** Cross-correlation between total lipid content summed of saturated (SAT), monounsaturated (MUFA) and polyunsaturated (PUFA) fatty acids, based on GC data

	Total lipid content	SAT	MUFA	PUFA
Total lipid content	1.000	− 0.0998	0.0053	0.0689
SAT	− 0.0998	1.000	− 0.7665	− 0.2307
MUFA	0.0053	− 0.7665	1.000	− 0.4391
PUFA	0.0689	− 0.2307	− 0.4391	1.000

## Additional file

**Additional file 1: Table S1.** Main lipid classes analysed by thin layer chromatography (TLC) for the yeast strains *Solicoccozyma terricola* 4517. *Lipomyces starkeyi* 1807. *Rhodotorula babjevae* 7808. *Rhodotorula toruloides* 14. *Rhodotorula glutinis* 5805 grown in pre-culture (P) and nitrogen limited media containing glucose (G), xylose (X) and mixture of glucose and xylose (M). The results are presented in percentage (%). **Table S2.** Main lipid classes analysed by thin layer chromatography (TLC) for the yeast strains *Solicoccozyma terricola* 4517. *Lipomyces starkeyi* 1807. *Rhodotorula babjevae* 7808. *Rhodotorula toruloides* 14. *Rhodotorula glutinis* 5805 grown in pre-culture (P) and nitrogen limited media containing glucose (G), xylose (X) and mixture of glucose and xylose (M). The results are presented in percentage (%) and standard deviation (STD).

## Data Availability

The yeast strains used in this study are available through culture collections. All data generated or analysed during this study are included in this published article (and its additioanal files).
