# Supplementary material for: Biochemical profiling, prediction of total lipid content and fatty acid profile in oleaginous yeasts by FTIR spectroscopy
Source: Biotechnol Biofuels. 2019 Jun 6;12:140. doi: 10.1186/s13068-019-1481-0 (PMC6551905; doi:10.1186/s13068-019-1481-0)
Supplement: Supplementary file 1 — Additional file 1: Table S1. Main lipid classes analysed by thin layer chromatography (TLC) for the yeast strains Solicoccozyma terricola 4517. Lipomyces starkeyi 1807. Rhodotorula babjevae 7808. Rhodotorula toruloides 14. Rhodotorula glutinis 5805 grown in pre-culture (P) and nitrogen limited media containing glucose (G), xylose (X) and mixture of glucose and xylose (M). The results are presented in percentage (%). Table S2. Main lipid classes analysed by thin layer chromatography (TLC) for the yeast strains Solicoccozyma terricola 4517. Lipomyces starkeyi 1807. Rhodotorula babjevae 7808. Rhodotorula toruloides 14. Rhodotorula glutinis 5805 grown in pre-culture (P) and nitrogen limited media containing glucose (G), xylose (X) and mixture of glucose and xylose (M). The results are presented in percentage (%) and standard deviation (STD). [file 13068_2019_1481_MOESM1_ESM.docx]

**Table S1** Main lipid classes analysed by thin layer chromatography (TLC) for the yeast strains *Solicoccozyma terricola* 4517. *Lipomyces starkeyi* 1807. *Rhodotorula babjevae* 7808. *Rhodotorula toruloides* 14. *Rhodotorula glutinis* 5805 grown in pre-culture (P) and nitrogen limited media containing glucose (G), xylose (X) and mixture of glucose and xylose (M)**.** The results are presented in percentage (%).

|  | **Phospho**  **lipids** | **Mono**  **glycerol** | **1.2 Di**  **glycerol** | **1.3 Di**  **glycerol** | **Sterols** | **Free fatty**  **acids** | **Triacyl**  **glycerols** |
| --- | --- | --- | --- | --- | --- | --- | --- |
| Solicoccozyma terricola 4517 (P) | 11.79±0.11 | 5.96±0.59 | 2.24±0.51 | 4.67±0.32 | 3.53±0.42 | 25.17±0.63 | 46.65±2.01 |
| Solicoccozyma terricola 4517 (G) | 7.9 ±0.4 | 5.51±0.54 | 3.12±0.28 | 3.94±0.36 | 3.86±0.51 | 12.11±0.4 | 63.53±0.56 |
| Solicoccozyma terricola 4517 (M) | 7.00 ±0.33 | 5.93±0.66 | 2.48±0.12 | 3.62±0.44 | 2.82±0.21 | 11.93±0.43 | 66.23±0.7 |
| Solicoccozyma  terricola 4517 (X) | 5.26 ±0.25 | 5.15±0.2 | 3.03±0.16 | 2.58±0.43 | 3.24±0.23 | 10.33±0.86 | 70.41±0.68 |
| Lipomyces  starkeyi 7544 (P) | 19.45±0.61 | 4.77±0.54 | 2.79±0.19 | 3.48±0.32 | 1.99±0.4 | 35.09±0.85 | 32.45±0.24 |
| Lipomyces  starkeyi 7544 (G) | 7.44±0.52 | 4.17±0.08 | 5.11±0.17 | 2.98±0.57 | 3.04±0.3 | 6.30±0.57 | 70.97±0.88 |
| Lipomyces  starkeyi 7544 (M) | 7.37±0.43 | 4.08±0.29 | 6.39±0.54 | 3.96±0.34 | 4.11±0.37 | 9.36±0.46 | 64.74±0.59 |
| Lipomyces  starkeyi 7544 (X) | 6.45±0.32 | 2.64±0.19 | 6.08±0.43 | 3.56±0.38 | 2.86±0.36 | 8.09±0.7 | 70.33±1.98 |
| Lipomyces  starkeyi 1807 (P) | 14.02±0.88 | 8.49±0.84 | 3.10±0.45 | 3.54±0.27 | 3.08±0.45 | 25.16±0.59 | 43.39±0.67 |
| Lipomyces  starkeyi 1807 (G) | 5.46±0.26 | 3.21±0.54 | 3.34±0.46 | 2.39±0.46 | 3.35±0.39 | 7.05±0.2 | 75.21±1.8 |
| Lipomyces  starkeyi 1807 (M) | 6.25±0.71 | 3.73±0.12 | 2.78±0.6 | 2.56±0.5 | 3.34±0.33 | 9.53±0.18 | 71.83±1.85 |
| Lipomyces  starkeyi 1807 (X) | 5.67±0.44 | 3.48±0.28 | 2.58±0.3 | 2.36±0.29 | 3.89±0.3 | 8.43±0.32 | 73.59±1.0 |
| Rhodotorula babjevae 7808 (P) | 22.49±1.18 | 1.81±0.35 | 2.99±0.32 | 5.06±0.04 | 1.02±0.14 | 41.00±0.5 | 25.90±0.89 |
| Rhodotorula babjevae 7808 (G) | 10.28±0.45 | 1.08±0.28 | 2.74±0.32 | 3.03±0.34 | 3.58±0.5 | 8.92±0.79 | 70.65±1.76 |
| Rhodotorula babjevae7808 (M) | 11.88±0.34 | 1.30±0.48 | 3.42±0.26 | 4.26±0.29 | 5.46±0.5 | 14.27±0.72 | 59.74±1.76 |
| Rhodotorula babjevae 7808 (X) | 10.41±0.23 | 0.82±0.12 | 2.56±0.14 | 3.74±0.23 | 6.17±0.27 | 20.04±0.19 | 56.47±0.46 |
| Rhodotorula toruloides 14 (P) | 18.91±0.63 | 1.71 | 2.26±0.06 | 4.21±0.49 | 5.93±0.04 | 41.36±0.31 | 26.49±0.39 |
| Rhodotorula toruloides 14 (G) | 6.18±0.28 | 0.82±0.02 | 2.19±0.11 | 2.09±0.15 | 4.85±0.06 | 9.16±0.04 | 74.73±0.35 |
| Rhodotorula toruloides 14 (M) | 6.97±0.13 | 0.88±0.2 | 1.76±0.22 | 1.96±0.05 | 4.87±0.14 | 9.78±0.52 | 73.79±0.91 |
| Rhodotorula toruloides 14 (X) | 7.90±0.19 | 1.11±0.2 | 1.59±0.23 | 1.57±0.37 | 5.70±0.14 | 12.46±0.45 | 69.69±0.92 |
| Rhodotorula glutinis 5805 (P) | 24.08±0.02 | 3.78±0.4 | 2.58±0.14 | 5.47±0.01 | 6.91±0.23 | 30.24±0.33 | 26.96±1.08 |
| Rhodotorula glutinis 5805 (G) | 7.04±0.21 | 0.8 | 1.86 | 2.04 | 5.19±0.01 | 14.07±0.07 | 71.37±2.2 |
| Rhodotorula glutinis 5805 (M) | 7.18±0.02 | 1±0.01 | 2.13±0.07 | 3.83±0.03 | 7.00±0.03 | 27.78±0.25 | 51.11±0.36 |
| Rhodotorula glutinis 5805 (X) | 14.59±0.09 | 0.81±0.14 | 2.01±0.09 | 2.62±0.16 | 7.67±0.25 | 22.91±0.27 | 49.40±0.14 |

**Table S2** Main lipid classes analysed by thin layer chromatography (TLC) for the yeast strains *Solicoccozyma terricola* 4517. *Lipomyces starkeyi* 1807. *Rhodotorula babjevae* 7808. *Rhodotorula toruloides* 14. *Rhodotorula glutinis* 5805 grown in pre-culture (P) and nitrogen limited media containing glucose (G), xylose (X) and mixture of glucose and xylose (M). The results are presented in percentage (%) and standard deviation (STD).

| Yeast strain | Phospho  lipids | Mono-glycerol | 1.2 Di  glycerol | 1.3 Di  glycerol | Sterols | Free fatty acids | TAG |
| --- | --- | --- | --- | --- | --- | --- | --- |
| *Solicoccozyma terricola* 4517 (P) | 11.79 | 5.96 | 2.24 | 4.67 | 3.53 | 25.17 | 46.65 |
| STD | 0.11 | 0.59 | 0.51 | 0.32 | 0.42 | 0.63 | 2.01 |
| *Solicoccozyma terricola* 4517 (G) | 7.93 | 5.51 | 3.12 | 3.94 | 3.86 | 12.11 | 63.53 |
| STD | 0.40 | 0.54 | 0.28 | 0.36 | 0.51 | 0.40 | 0.56 |
| *Solicoccozyma terricola* 4517 (M) | 7.00 | 5.93 | 2.48 | 3.62 | 2.82 | 11.93 | 66.23 |
| STD | 0.33 | 0.66 | 0.12 | 0.44 | 0.21 | 0.43 | 0.70 |
| *Solicoccozymaterricola* 4517 (X) | 5.26 | 5.15 | 3.03 | 2.58 | 3.24 | 10.33 | 70.41 |
| STD | 0.25 | 0.20 | 0.16 | 0.43 | 0.23 | 0.86 | 0.68 |
| *Lipomyces starkeyi* 1807 (P) | 14.02 | 8.49 | 3.10 | 3.54 | 3.08 | 25.16 | 43.39 |
| STD | 0.88 | 0.84 | 0.45 | 0.27 | 0.45 | 0.59 | 0.67 |
| *Lipomyces starkeyi* 1807 (G) | 5.46 | 3.21 | 3.34 | 2.39 | 3.35 | 7.05 | 75.21 |
| STD | 0.26 | 0.54 | 0.46 | 0.46 | 0.39 | 0.20 | 1.80 |
| *Lipomyces starkeyi* 1807 (M) | 6.25 | 3.73 | 2.78 | 2.56 | 3.34 | 9.53 | 71.83 |
| STD | 0.71 | 0.12 | 0.60 | 0.50 | 0.33 | 0.18 | 1.85 |
| *Lipomyces starkeyi* 1807 (X) | 5.67 | 3.48 | 2.58 | 2.36 | 3.89 | 8.43 | 73.59 |
| STD | 0.44 | 0.28 | 0.30 | 0.29 | 0.30 | 0.32 | 1.00 |
| *Rhodotorula babjevae* 7808 (P) | 22.49 | 1.81 | 2.99 | 5.06 | 1.02 | 41.00 | 25.90 |
| STD | 1.18 | 0.35 | 0.32 | 0.04 | 0.14 | 0.50 | 0.89 |
| *Rhodotorula babjevae* 7808 (G) | 10.28 | 1.08 | 2.74 | 3.03 | 3.58 | 8.92 | 70.65 |
| STD | 0.45 | 0.28 | 0.32 | 0.34 | 0.50 | 0.79 | 1.76 |
| *Rhodotorula babjevae* 7808 (M) | 11.88 | 1.30 | 3.42 | 4.26 | 5.46 | 14.27 | 59.74 |
| STD | 0.34 | 0.48 | 0.26 | 0.29 | 0.50 | 0.72 | 1.76 |
| *Rhodotorula babjevae* 7808 (X) | 10.41 | 0.82 | 2.56 | 3.74 | 6.17 | 20.04 | 56.47 |
| STD | 0.23 | 0.12 | 0.14 | 0.23 | 0.27 | 0.19 | 0.46 |
| *Rhodotorula toruloides* 14 (P) | 18.91 | 1.71 | 2.26 | 4.21 | 5.93 | 41.36 | 26.49 |
| STD | 0.63 | 0.00 | 0.06 | 0.49 | 0.04 | 0.31 | 0.39 |
| *Rhodotorula toruloides* 14 (G) | 6.18 | 0.82 | 2.19 | 2.09 | 4.85 | 9.16 | 74.73 |
| STD | 0.28 | 0.02 | 0.11 | 0.15 | 0.06 | 0.04 | 0.35 |
| *Rhodotorula toruloides* 14 (M) | 6.97 | 0.88 | 1.76 | 1.96 | 4.87 | 9.78 | 73.79 |
| STD | 0.13 | 0.20 | 0.22 | 0.05 | 0.14 | 0.52 | 0.91 |
| *Rhodotorula toruloides* 14 (X) | 7.90 | 1.11 | 1.59 | 1.57 | 5.70 | 12.46 | 69.69 |
| STD | 0.19 | 0.20 | 0.23 | 0.37 | 0.14 | 0.45 | 0.92 |
| *Rhodotorula glutinis* 5805 (P) | 24.08 | 3.78 | 2.58 | 5.47 | 6.91 | 30.24 | 26.96 |
| STD | 0.02 | 0.40 | 0.14 | 0.01 | 0.23 | 0.33 | 1.08 |
| *Rhodotorula glutinis* 5805 (G) | 7.04 | 0.80 | 1.86 | 2.04 | 5.19 | 14.07 | 71.37 |
| STD | 0.21 | 0.00 | 0.00 | 0.00 | 0.01 | 0.07 | 2.20 |
| *Rhodotorula glutinis* 5805 (M) | 7.18 | 1.00 | 2.13 | 3.83 | 7.00 | 27.78 | 51.11 |
| STD | 0.02 | 0.01 | 0.07 | 0.03 | 0.03 | 0.25 | 0.36 |
| *Rhodotorula glutinis* 5805 (X) | 14.59 | 0.81 | 2.01 | 2.62 | 7.67 | 22.91 | 49.40 |
| STD | 0.09 | 0.14 | 0.09 | 0.16 | 0.25 | 0.27 | 0.14 |
